# Current Treatment of Stress Urinary Incontinence by Bulking Agents and Laser Therapy—An Update

**DOI:** 10.3390/jcm13051377

**Published:** 2024-02-28

**Authors:** Michal Sikora, Marianne Gamper, Irena Zivanovic, Julia Münst, Helena Bischofberger, Jacek Kociszewski, Volker Viereck

**Affiliations:** 1 Department of Gynecology and Obstetrics, Spital Thurgau Frauenfeld, 8501 Frauenfeld, Switzerland; michal.sikora@stgag.ch (M.S.); marianne.gamper@stgag.ch (M.G.); irena.zivanovic@stgag.ch (I.Z.); julia.muenst@stgag.ch (J.M.); helena.bischofberger@stgag.ch (H.B.); 2 Department of Gynecology and Obstetrics, Evangelisches Krankenhaus Hagen-Haspe, 58135 Hagen, Germany; kociszewski@evk-haspe.de

**Keywords:** stress urinary incontinence, bulking agent, Bulkamid^®^, Macroplastique^®^, Urolastic^®^, Erbium:YAG laser, CO_2_ laser, intravaginal, intraurethral, randomized controlled trial

## Abstract

Stress urinary incontinence (SUI) affects around 20% of women. In addition to the established suburethral sling insertion, two less invasive approaches are of interest today: urethral bulking agents and vaginal laser therapy. This review discusses articles through December 2023 identified by a PubMed literature search using the keywords “incontinence” and “bulking” or “laser”. Although the two approaches are less effective than sling insertions, there are specific conditions in which one or the other technique is more advantageous. Injecting bulking agents into the urethra only takes some minutes and works without general anesthesia. The method is particularly suited for elderly, frail, or obese patients with multiple comorbidities, but is also applicable for all patients and in combination with other therapies. Generally, the safety profile is good but differs between bulking materials. Two laser types—the Erbium:YAG laser with SMOOTH-mode and the fractional ablative CO_2_ laser—deliver heat into the tissue to induce tissue tightening and regeneration. Intravaginal laser therapy improves mild to moderate SUI, while studies describe how intraurethral laser therapy is also beneficial for severe SUI. Young women between childbirths, as well as postmenopausal women, may benefit from laser therapy. The method is safe, can be performed on an outpatient basis, and does not require any artificial material.

## 1. Introduction

According to recent data, more than 40% of all women suffer from urinary incontinence [1]. However, for many the topic is too embarrassing to talk about. It often takes a long time before professional help is sought.

Stress urinary incontinence (SUI), i.e., the involuntary leakage of urine during physical exertion such as lifting weights, climbing stairs, sports, or when coughing, sneezing, or laughing, is the most common form of incontinence (48%), followed by mixed urinary incontinence (MUI) (34%) and urge incontinence (17%) [2]. SUI tends to dominate among younger women while the numbers of women with urge incontinence (UUI) and MUI increase with age [1]. The reason for stress incontinence is the inability of the closure system to withstand the sudden increase of the bladder pressure. Urethral or pelvic floor muscles that react too weakly or too slowly, a weak connective tissue and thin mucous membranes caused by postmenopausal estrogen deficiency, or the relaxation of urethral ligaments, can lead to urethral closure insufficiency [3]. Depending on the severity, urine may be lost in drops, splashes, or in a stream. Risk factors for stress incontinence include age, pregnancy and childbirth, obesity, smoking, chronic cough, or constipation [4].

Conservative treatment is the recommended first-line therapy for SUI. Weight reduction or cessation of smoking can improve incontinence. Pessary therapy and/or local or systemic estrogen treatment may also help. Pelvic floor physiotherapy is very successful [5]. Targeted training can build pelvic floor muscles, and voluntary pre-contraction of pelvic floor muscles immediately before a sudden abdominal pressure increase can prevent urine leakage. Complementary whole body vibration therapy can additionally stimulate reflexive muscle contractions. This leads to increased pelvic muscle tension at rest, improved contractility of the pelvic floor muscles, and to improved continence [6].

Generally, surgery is recommended after three months of unsuccessful conservative therapy [7]. The application of local estrogen before the intervention helps to improve tissue quality [8]. Surgery can be performed earlier if the patient is unwilling or unable to perform conservative therapy [9].

## 2. Surgical Therapies of SUI

Today, the gold standard surgical therapy for SUI is the insertion of a retropubic suburethral sling (“tension-free vaginal tape”, TVT) [3]. Cure rates are as high as 90 percent [10]. Transobturator tapes (TOTs) are similarly as effective as TVTs in the short and medium term [11,12,13]. However, TVTs are becoming more popular due to better long-term data and fewer postoperative complications [14]. TOTs are more often associated with groin pain and dyspareunia [8,11,12]. The use of mini slings could not become established and long-term data are missing [8,11]. Colposuspension, the previous standard treatment for SUI, has similarly high cure rates [15]. This technique is much more invasive and is rarely used today [16,17], with the exception of specialized centers that offer colposuspension as an efficient mesh-free alternative [18].

Warnings from the U.S. Food and Drug Administration (FDA) on the use of vaginal synthetic mesh in descensus surgery (2008, 2011, 2016) led to the discontinuation of synthetic incontinence slings in certain countries (England, Scotland, New Zealand) since 2017/2018 [19]. However, in Germany, Austria, and Switzerland, mid-urethral synthetic slings are still allowed without restrictions. Nevertheless, the need for surgical alternatives is also high in these countries. Two less invasive approaches are of increasing interest today, namely bulking agents and laser therapy. This review discusses articles through December 2023 identified by a PubMed literature search using the keywords “incontinence” and “bulking” or “laser”.

## 3. Bulking Agents to Treat SUI

**Figure 1:**
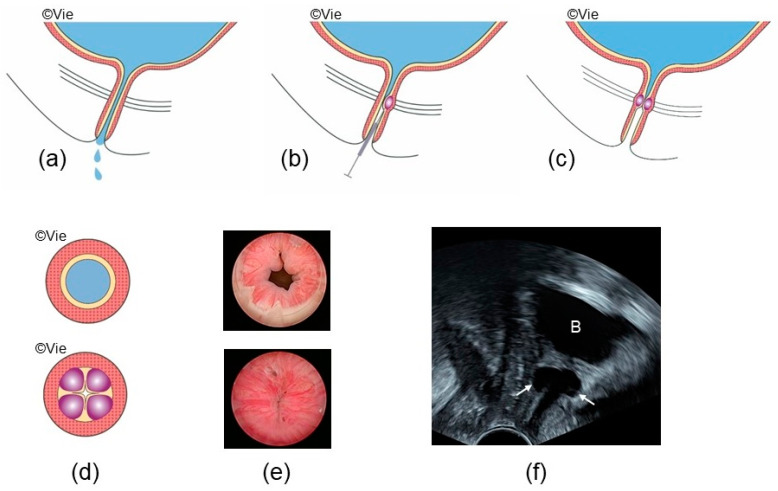
Bulking agents injection: (**a**) Stress urinary incontinence; (**b**) Transurethral injection of bulking agents; (**c**) Continence after bulking agents injection; (**d**,**e**) Top: open urethra (funneling), incontinence; bottom: coaptation after injection of four depots of bulking agents, continence; (**e**) Urethroscopy before and after Bulkamid^®^ injection; (**f**) Pelvic floor sonography, sagittal view: Bulkamid^®^ depots (white arrows) are visible. B: bladder.

A minimal invasive option for the therapy of stress incontinence is the injection of a bulking agent into the urethra (Figure 1). This therapeutic technique has been known for almost 100 years. Many different bulking agents were used. However, most of them have been withdrawn from the market due to lack of efficacy or for material-related complications which is even worse. Some adverse events were very serious, such as allergies, migration, erosions, fistula formation, sterile abscesses, and infections [20]. 

Over the years, new bulking materials entered the market. Materials and injection techniques have developed and improved significantly. Since the FDA warning on vaginal meshes and the associated pause on sling insertion in England [19], the use of bulking agents increased. Available today are Bulkamid^®^, Macroplastique^®^, Durasphere^®^, Coaptite^®^, and Urolastic^®^. The first four materials have FDA approval for the treatment of SUI [21].

There are two classes of bulking material, the **particulate bulking agents** such as Macroplastique^®^, Durasphere^®^, Coaptite^®^, and Urolastic^®^ that become solid after trans- or periurethral injection, and the **homogeneous gel-type bulking agents** (for example, Bulkamid^®^) that remain flexible [22,23]. The surrounding tissue reacts with agents of both classes, ideally resulting in a stable and permanent integration of the bulking agent in the urethral submucosa. By this approach, coaptation of the urethra is improved, the urethral resistance at rest is increased, and continence is achieved [22] (Figure 1). The most serious adverse events of the five bulking agents are summarized (Table 1) [21,22].

Recent publications mostly describe studies on Bulkamid^®^, Macroplastique^®^, or Urolastic^®^, which are the three bulking agents marketed in Europe [24]. In the following, study outcomes are discussed.

### 3.1. Urolastic^®^

Urolastic^®^ has had CE approval since 2009 and is marketed in Europe [24]. The two components of Urolastic^®^ (polydimethylsiloxane and a cross-linking agent) are mixed and injected periurethraly with the guidance of an applicator but without urethroscopic view. Ideally, four depots of 0.8 mL each are placed around the urethra. There, the material polymerizes within 3 to 4 min into a solid rubberlike implant [25]. Bulking depots are to be positioned in a plane at the mid-urethra [25], and the shape of the implant ideally should be spherical to reduce the risk of pain from sharp extensions [25]. It seems very difficult for most physicians to perform this blind injection technique with “acceptable failure rates” [26]. Only two out of five physicians reached a level of competence after 20 or 40 injections, respectively [26]. Computer tomography imaging of Urolastic^®^ implants showed that 45% of all implants were not located at the intended position, and in 23% of all injections, the implant was scattered rather than spherical [25]. Scattered implants may follow anatomical structures such as small blood vessels, and hence are difficult to remove by surgery [25,27]. The outcome after Urolastic^®^ injection was 15% with no improvement at all, 45% with 20–90% improvement, and 40% with >90% improvement of SUI. Post-operative pain was reported by 45% of the patients, and for 35%, removal of the bulking material was necessary [25]. Adverse events reported after Urolastic^®^ injection were 9.9% dyspareunia, 23.6% exposure through the vaginal wall, and 19.8% surgical excisions [28]. Another study reported 25.8% overall complications with 9.1% of the solidified material in the injection channel, 4.5% displaced oval-shaped material floating in the bladder, and 6.1% displaced material under the urethra causing pain and dyspareunia [29]. The displaced material could have ended up in the wrong place due to migration or a misdirected (blind) initial injection (Table 1) [29,30,31]. In another study, erosion through the anterior vaginal wall and postoperative pain led to 18% or 26% (partial) removal of the bulking material under local or general anesthesia [30]. In contrast to Bulkamid^®^ and Macroplastique^®^, Urolastic^®^ material creates shadows that clearly limits ultrasonographic visibility on examination [27]. Therefore, functional sonography to control the position and the size of the implants is difficult, which has negative implications for complication management. The serious complications of this product require close surveillance in the future.

**Table 1:** Complications of different bulking materials.

Complication ^1^	Carbon-Coated Zirconium Oxide	Calcium Hydroxylapatite	Polydimethyl- Siloxane Polymer	Polydimethyl- Siloxane	Polyacrylamide Hydrogel
	Durasphere^®^	Coaptite^®^	Urolastic^®^	Macroplastique^®^	Bulkamid^®^
Migration	**Yes**	**Yes**	**Yes ^2^**		
Granuloma		**Yes**			
Sterile abscesses (pseudo-cysts)	**Yes**				
Erosion		**Yes**	**Yes**	**Yes**	
Calcification					
Allergies					
Injection site rupture					**Yes**

^1^: Summarized information from [21,22]. ^2^: Migration or initial injection at unintended place [29,30,31]. Trade names are shown in purple font. Reported complications are highlighted in red.

### 3.2. Macroplastique^®^

Macroplastique^®^ received CE approval in 1991 and FDA approval in 2006 for use in treating adult women diagnosed with SUI primarily due to intrinsic sphincter deficiency (ISD) [24]. Macroplastique^®^ is a non-biodegradable agent, consisting of polydimethylsiloxane (silicone) macroparticles suspended in a water-soluble absorbable carrier gel (polyvinylpyrolidone) to facilitate injection. Injection of silicone can be accomplished with standard cystoscopic instrumentation transurethraly or with a blind approach using an applicator. Two or three large deposits with a total volume of 5 mL are injected at the mid-urethral level [32,33]. Based on transperineal ultrasonography, Macroplastique^®^ slightly decreased in volume over time, but did not change in configuration or position throughout several years [33]. The large size of the silicone particles (>100 μm) should reduce the risk of migration. The material is inert and only triggers a minimally inflammatory reaction without an antibody-driven immune response [33]. Histology shows fibroblast rearrangement around the particles and encapsulation with collagen, resulting in firm nodules six weeks after implantation [32]. In a multicenter prospective study analyzing patients treated with Macroplastique^®^ after recurrent SUI, subjective and objective cure rates at the 3-year follow-up were 81% and 83%, respectively [34]. Bulking failed in 10.6% and patients received further incontinence surgery. The complication rate was 8.5% (4/47), two of the patients had de novo overactive bladder syndrome, and two had a persisting voiding dysfunction [34]. Reports of serious adverse events from Macroplastique^®^ are uncommon but have been described [35]. Urge or stress incontinence, recurrent urinary tract infections, and urinary urgency or frequency may indicate urethral or bladder exposures [35,36]. Erosions can occur even years after injection, and most surgical removal led to recurrent SUI (Table 1) [35]. A systematic review and meta-analysis reported an 84% cure and improvement rate for Macroplastique^®^ after failed sling insertion [37]. A second injection may be required to achieve the desired success in about 30–40% of all cases [33,38,39].

### 3.3. Bulkamid^®^

Bulkamid^®^ received CE approval in 2006 and FDA approval in 2020 for patients with SUI or stress-dominant MUI [24]. Bulkamid^®^ is a non-particulate homogeneous polyacrylamide hydrogel composed of 2.5% cross-linked polyacrylamide and 97.5% water. The originally injected filling volume remains constant and is integrated into the tissue over time without hardening.

**Figure 2:**
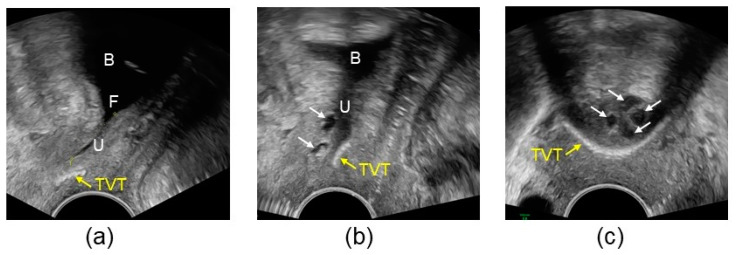
Pelvic floor sonography: Combined effect of bulking agent (Bulkamid^®^) and a sling. (**a**) Recurrence after TVT insertion, situation before Bulkamid^®^ injection. In the sagittal view, funneling is visible at straining, the position of the TVT is too distal; (**b**) Continence after subsequent Bulkamid^®^ insertion. At straining, the Bulkamid^®^ depot is pressed towards the TVT. Together, they achieve urethral continence; (**c**) Axial view, same patient: the four Bulkamid^®^ depots are localized in one plane around the urethra to render continence. Due to coaptation, the urethral lumen itself is closed while funneling is no longer present. B: bladder; F: funneling; U: urethra; white arrows point to Bulkamid^®^ depots; yellow arrows point to TVT.

Bulkamid^®^ has numerous ideal properties for a bulking agent. It is biocompatible, non-biodegradable, non-absorbable, non-migratory, non-toxic, and hypoallergenic, does not induce inflammation, and can be easily injected [40]. Today, mostly four rather than three depots of 0.5 mL each are injected in the proximal third of the urethra under urethroscopic view [41,42] (Figure 1). The depots are ideally arranged circularly on the same level to close the urethral lumen (coaptation) (Figure 1 and Figure 2). The procedure takes only a few minutes, can be performed under local anesthesia, and does not require one to stop anticoagulation. Bulkamid^®^ is safe, with only a low risk of product-related complications. The most common side effects are acute urinary retention (0–20%), urinary tract infections (1.6–40%), and de novo urgency symptoms (0–10%) [21,43]. Erosions are extremely rare or undocumented. Rarely, a Bulkamid^®^ depot may leak (Table 1) [22], or the depot is hypermobile and lacks tissue support. Depots placed too high on the bladder neck can close the urethra like a valve and must be surgically removed in extremely rare cases. A systematic review and meta-analysis reported an 80% cure and improvement rate for Bulkamid^®^ after failed sling insertion [37]. Re-injection rates to improve continence varied significantly between studies, ranging from 0% to 77% [42,44,45], while the median time between injections was 3 months [45].

### 3.4. Bulkamid^®^ Therapy—The New Gold Standard?

Is Bulkamid^®^ the new gold standard of surgical therapy for stress incontinence? Peter Dwyer mentioned this possibility for the first time in an “Editorial Comment” to the randomized controlled trial (RCT) that compared Bulkamid^®^ vs. TVT [46]. This may be true for England or other countries where TVT insertions are no longer performed. However, the RCT clearly showed that TVT insertion was superior to Bulkamid^®^ injection in the primary situation [46]. Precisely 83.1% of the patients were cured 3 years after TVT insertion, whereas only 33.3% were cured after Bulkamid^®^ injection (per-protocol-analysis). In the Bulkamid^®^ arm, 36% received a second Bulkamid^®^ injection in the 3 years following the first intervention, and 31% even had a TVT. In the TVT arm, the TVT insertion was sufficient for 96%, only four patients received a second incontinence surgery, one (1%) a second TVT insertion, and three (3%) a Bulkamid^®^ injection [46]. Evaluation of subjective quality of life and sexual function showed improvements in both arms, again with better outcomes for the TVT group [47]. Even in the intention-to-treat analysis, patient satisfaction (VAS ≥ 80) at 3 years was significantly higher in the TVT group (94.6%) than in the Bulkamid^®^ group (67.7%) [46]. Complications were higher in the TVT arm, with 6.7% bladder perforations and 3.3% vaginal tape erosions. The rate of corrective surgery was 6.5% in the TVT group and 2.1% in the Bulkamid^®^ group [46]. 

### 3.5. Bulking Agents or Midurethral Slings to Cure SUI

Because of the mesh discussion, many other recent publications such as reviews, retrospective registry/database evaluations, and surveys compared bulking agents with other surgical procedures for SUI. In all cases, the sling operation was superior or favored over bulking injection. A systematic review of publications from 2000 to 2021 showed that bulking agents were subjectively less effective than surgical procedures and that there was no statistically significant difference regarding safety [48]. Another review found that mid-urethral slings are the preferred second-line surgical therapy after failed sling surgery and that bulking agents have a lower durability and efficacy than other treatments [49]. In a retrospective cohort study of data between 2001 and 2018 from a U.S. database, patients were identified that received a prolapse surgery together with an operative incontinence treatment. In the two postoperative years, urethral bulking was associated with a higher rate of repeat procedure and more complications than mid-urethral sling insertion [50]. In a retrospective cohort study of data between 2001 and 2017 from a Norwegian database, patients with surgical treatment of SUI were identified. Within five postoperative years, the proportion of patients without surgery for recurrent SUI was 97.7% for retropubic slings, 96.1% for obturator slings, and 72.4% for Bulkamid^®^ injections [51]. Data from the National Health Service (NHS) in England between 2012 and 2018 showed a decrease in mid-urethral sling surgery and an increase in bulking procedures [18]. An electronic survey of the members of the International Urogynecological Association (IUGA) and the American Urogynecologic Society (AUGS) showed that retropubic mid-urethral slings were the preferred initial surgical treatment for SUI (62%), whereas bulking agents were only preferred by 5% of the survey responders [52]. Another survey among healthcare professionals in England showed that using mid-urethral slings for treating SUI was still the most popular choice before and after informing of specific complications [53]. After informing patients with primary SUI about efficacy, surgery, and side effects of midurethral slings and bulking agents, patients considered bulking agents a valuable treatment option even though it had lower cure rates than sling surgery [54]. 

### 3.6. Indication for Bulking Agent Therapy

Even though most studies favor mid-urethral slings over bulking agents, there are certain situations where bulking agent therapy is preferred. Traditionally, bulking agents were used for women with bothersome SUI due to ISD [23]. Bulking therapy is also suitable in patients with an immobile or rigid urethra, e.g., after colposuspension [8]. Urodynamics and pelvic floor ultrasonography are particularly important for this diagnosis. Due to low complication rates, short operative times, and no need to stop anticoagulation, Bulkamid^®^ injection is the treatment of choice for elderly and obese women with multiple comorbidities and previous uro-/gynecologic surgery, reduced anesthetic ability, or anticoagulation [8]. A retrospective dataset analysis of women over 65 years showed that patients receiving bulking agents were notably sicker and have a shorter life expectancy compared to those undergoing sling insertion, suggesting these factors guide patient selection [55]. Bulking therapy is more successful for patients with mild SUI [45,56,57] or with ISD [58]. Patients younger than 60 years preferred an office setting rather than an operation theatre [59]. However, in a frail and elderly patient population with comorbidities, a stationary setting is recommended to closely monitor post-void residual urine and post-operative voiding [42].

Bulking can be performed to treat recurrent SUI, either after sling failure (Figure 2) [34,37,43,60] or after ineffective primary bulking [61]. Re-bulking mostly with Bulkamid^®^ improved median twenty-four-hour pad-weight from 90 g to 35 g and achieved a dry rate of 36% [61]. In women with recurrent SUI/MUI after sling insertion (salvage population), Bulkamid^®^ injection achieved very good results with cure/improvement rates of 83.6% at 12 months [43]. Bulking can be performed after sling/bulking, and if ineffective, further bulking injections or sling insertions are possible [39,46]. Continence is achieved by the interaction of primary and secondary incontinence treatment; for example, with the joint support of the urethra by the sling and the bulking depot (Figure 2). Functional imaging with pelvic floor sonography is crucial to plan the surgical strategy and to control outcome, position, size, and number of the implants.

Bulkamid^®^ is not a short-term therapy and does not require frequent repeat injection. Long-term data with 7–8-year follow-ups are available. Cure/improvement rate was 65.2% [62], and subjective success rate was 53% [45]. Sonographically, Bulkamid^®^ depots could still be detected after 7 years [63]; i.e., if properly positioned, Bulkamid^®^ does not need to be re-injected.

Bulking agents can also be used to treat MUI after failed sling insertion. Preoperative MUI was cured in 39% [60] or in 38.9% [43] of the patients. Both the stress and the urgency component of MUI can also be individually targeted by combining Bulkamid^®^ and Botox^®^ injections in a single operation [42]. At the 12-month follow-up, the objective cure rate for MUI was 50%. Within the first postoperative year, no patient required reinjection of the bulking agent and only 4% needed a second Botox^®^ injection [42]. Although the immediate retention rate requiring intermittent catheterization was 22%, retention was transient and resolved within the first postoperative days [42].

### 3.7. Conclusion—Bulking Agent Therapy

The bulking agents available today—particularly Bulkamid^®^ and Macroplastique^®^—for the treatment of SUI are effective and safe, while Urolastic^®^ has an inferior safety profile that requires close monitoring. The success rate after bulking agent injection is lower than after sling insertion. Bulkamid^®^ can be used as a primary therapy, but the outcome can be insufficient so that in over 60% of all cases a second intervention might be necessary. Patients with ISD or with an immobile urethra benefit from bulking injection rather than from a sling. Bulkamid^®^ injection is also successful in secondary therapy for recurrent SUI/MUI after sling, bulking, or colposuspension failure. If primary bulking is insufficient, continence can be improved by re-bulking or most efficiently by a subsequent sling insertion.

Urethral bulking agent injection is an ideal treatment option for frail, elderly, or obese women with multiple comorbidities. The bulking procedure only requires a brief surgery time and can be performed without discontinuation of anticoagulation and without general anesthesia.

## 4. Laser Therapy to Treat SUI

Laser therapy is another new and even less invasive option to treat SUI. It can be performed in an ambulatory setting and needs about 3–5 sessions at intervals of approximately one month [64]. One session takes about 15 to 30 min. Studies demonstrate subjective and objective cure/improvement rates of over 70% six months after the end of therapy. Complete cure (no urine loss) occurs in approximately 30% of patients [65]. The effect is transient and lasts for up to 1 to 2 years [64]. Refreshing treatments (top-ups) are possible. If there is no visible success after the first or second laser session, an alternative therapy should be considered.

Laser therapy is a new treatment option for SUI. Laser treatments for medical specialties including gynecology are FDA approved, but not specifically for SUI [65,66]. For SUI, the Fotona SMOOTH laser treatment has clearance from Canada, Europe, Australia, Taiwan, and Singapore. Since 2012 [67,68], the number of publications increased drastically. Retrospective and prospective original articles, RCTs, reviews and meta-analyses were published, and laser therapy was evaluated in expert opinions and guidelines [7,69,70]. The latest findings and publications are summarized below.

### 4.1. Laser Types and Mechanism of Action

**Figure 3:**
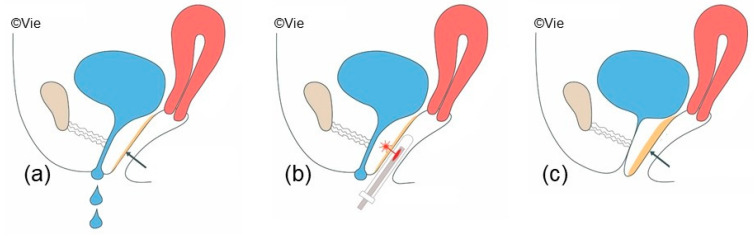
Intravaginal laser therapy: (**a**) Incontinence, weak anterior vaginal wall without sufficient support of the urethra; (**b**) Laser treatment of the anterior vaginal wall with an angular probe; (**c**) Continence after tightening of the anterior vaginal wall. The arrows point to the anterior vaginal wall.

Laser procedures to treat SUI involve the insertion of a probe into the vagina (Figure 3). The laser energy is delivered by retracting the probe in approximately 5 mm steps from proximal to distal. Different adapters and handpieces direct the laser energy circularly (360°) to treat the entire vagina, or unidirectionally (90°) to only treat the anterior vaginal wall supporting the urethra.

Mainly two types of lasers with different wavelengths are used to treat SUI, the Erbium:YAG laser (2940 nm) and the CO_2_ laser (10,600 nm). The laser energy of the Erbium:YAG laser is delivered into the tissue by the “non-ablative” SMOOTH-mode technology [71]. Special pulse sequences heat the mucous tissue to 60 °C without damaging the superficial epithelium [71,72].

The “fractional micro-ablative” laser procedure was mostly performed with a CO_2_ laser, with a few using the fractional ablative mode of the Erbium:YAG laser [73]. Special laser pulses create fine micro-channels and micro-wounds in the tissue, through which heat can penetrate into deeper tissue layers (200–500 μm) [74]. This technique was initially used to treat atrophy in postmenopausal women with genitourinary syndrome of menopause (GSM), while subgroup analyses also showed an improvement in their urinary symptoms [75]. 

The two laser treatments trigger a photo-thermal effect on the vaginal wall. Elevated temperatures denature the highly organized triple helix structure of collagen, and lead to collagen contraction into thicker and shorter fibers and consequently to the induction of neo-collagenesis. This collagen tightening improves thickness, elasticity, and firmness of the vaginal wall, improves the suburethral support, and enhances urinary continence [76]. Further effects are a thickening of the glycogen enriched epithelium, vasodilatation, and neo-vascularization with improved blood flow in the lamina propria [74,77,78].

Alternatively or in addition to intravaginal application, the Erbium:YAG SMOOTH-mode treatment can also be applied directly in the urethra by using a special intraurethral device [79,80,81,82]. Based on the similarity between vaginal and urethral mucosa, laser treatment of the urethra is assumed to induce similar changes [79]. A study with healthy beagle dogs compared urethral Erbium:YAG treatment (SMOOTH-mode) with sham-urethral treatment and showed an increase in epithelium thickness and collagen expression only in the laser group [83]. By urethroscopy, more mucosal folds were visible in the laser group [83].

While both lasers induce thermal effects in the tissue, fractional micro-ablative CO_2_ lasers cause micro-wounding that initiates a regenerative healing process with edema and granulation formation followed by neo-collagenesis. The non-ablative Erbium:YAG laser uses mild hyperthermia to promote vasodilation, neo-angiogenesis, and neo-collagenesis. The Erbium:YAG laser is associated with milder post-operative discomfort, erythema, and edema, and the overall regeneration time is faster as compared to the CO_2_ laser [76]. Up to date, an RCT that directly compares the two laser therapies does not exist.

### 4.2. RCTs Investigating Laser Therapy for SUI

Eight RCTs investigated laser therapy for SUI, three of them with the Erbium:YAG laser with SMOOTH-mode settings [84,85,86], and five with the fractional CO_2_ laser (Table 2). Six RCTs evaluated laser vs. placebo (sham) control, one study had a third arm to test radiofrequency [87], and one RCT compared laser with pelvic floor physical training [86] and another with promestriene and lubricant [75]. Subjective study outcome analysis was often based on the International Consultation on Incontinence Questionnaire-Urinary Incontinence Short Form (ICIQ-UI SF) score, and objective outcomes were often analyzed by the one-hour pad weight test. In most studies, the subjective ICIQ-UI SF score significantly improved after laser therapy [75,84,87,88,89], and the objective pad weight significantly decreased after laser therapy (Table 2) [85,86,87,88]. Laser was significantly better than sham treatment in 50% (3/6) of the studies regarding the ICIQ-UI SF score [84,87,88], and in 75% (3/4) of the studies regarding the pad weight [85,87,88]. No significant outcome differences were found between laser and promestriene [75], laser and radiofrequency [87], or laser and pelvic floor physical training (Table 2) [86].

In three RCTs, the ICIQ-UI SF score was not significantly different after laser than after sham treatment [85,89,90]. This could be due to a placebo effect of the sham treatment. Among other study-dependent reasons, the application of an inactive sham probe with a permanent contact to the vaginal wall may already have a certain therapeutic effect.

**Table 2:** RCTs comparing laser therapy with sham or other interventions in treating female SUI.

RCTs	Number of Arms	Study Arms			Laser	Sessions	Follow-Up after Last Intervention	Number of Study Centers
Blaganje 2018 [84]	2	Non-ablative Er:YAG laser SMOOTH-mode vs. sham			Fotona IncontiLase	1	3 months	monocenter
Aguiar 2020 [75]	3	CO_2_ laser vs. promestriene vs. lubricant			Deka MonaLisa Touch	3	2 weeks	monocenter
Alexander 2022 [90]	2	CO_2_ laser vs. sham			Lumenis FemTouch	3	3 months	2 centers
Seki 2022 [87]	3	CO_2_ laser vs. radiofrequency vs. sham			Alma Femilift	3	12 months	monocenter
Lauterbach 2022 [88]	2	CO_2_ laser vs. sham			Lumenis FemTouch	1	3 months	monocenter
O’Reilly 2023 [85]	2	Non-ablative Er:YAG laser SMOOTH-mode vs. sham			Fotona IncontiLase	2	6 months	7 centers
Temtanakitpaisan 2023 [89]	2	CO_2_ laser +PFMT vs. sham+PFMT			Deka MonaLisa Touch	4	3 months	monocenter
da Fonseca 2023 [86]	2	Non-ablative Er:YAG laser SMOOTH-mode vs. PFMT			Fotona IncontiLase	3	12 months	monocenter
**RCT**	**Inclusion**							
Blaganje 2018 [84]	Premenopausal (35–65 years), sexually active, at least one vaginal delivery, SUI							
Aguiar 2020 [75]	Postmenopausal women over 50 years with GSM and SUI							
Alexander 2022 [90]	Pre- and postmenopausal women (34–79 years) with symptomatic and objective SUI							
Seki 2022 [87]	Pre- and postmenopausal women with SUI							
Lauterbach 2022 [88]	Pre- and postmenopausal women with previous successful CO_2_ laser treatment due to SUI							
O’Reilly 2023 [85]	Pre- and postmenopausal women with urodynamic SUI and at least one unsuccessful conservative SUI treatment							
Temtanakitpaisan 2023 [89]	Pre- and postmenopausal women with SUI or stress-dominant MUI							
da Fonseca 2023 [86]	Postmenopausal women with mild to moderate SUI							
**RCTs**	**Subjective Outcome**	**Laser BL vs. FU**	**Laser vs. Sham at FU**	**Laser vs. Control at FU**	**Objective Outcome**	**Laser BL vs. FU**	**Laser vs. Sham at FU**	**Laser vs. Control at FU**
Blaganje 2018 [84]	ICIQ-UI SF	**<0.001**	**<0.001**					
Aguiar 2020 [75]	ICIQ-UI SF	**0.004**		0.116 (promestriene)				
Alexander 2022 [90]	ICIQ-UI SF	?^1^	0.39		24 h Pad	?^2^	0.8	
Seki 2022 [87]	ICIQ-UI SF	**<0.001**	**0.019**	not sign (RF)	1 h Pad	**sign**	**0.03**	not sign (RF)
Lauterbach 2022 [88]	ICIQ-UI SF	**sign**	**0.003**		1 h Pad	**sign**	**<0.0001**	
O’Reilly 2023 [85]	ICIQ-UI SF	?^3^	0.063		1h Pad	**sign**	**<0.001**	
Temtanakitpaisan 2023 [89]	ICIQ-UI SF	**sign**	0.8281					
da Fonseca 2023 [86]					1 h Pad	**sign**		not sign (PFMT)

?^1^: data on significance not available, mean ICIQ-UI SF at BL: 13.47, mean ICIQ-UI SF at 3-month FU: 10.95 [90]. ?^2^: data on significance not available, median pad weight at BL: 16.5 mL, median pad weight at 3-month FU: 8 g [90]. ?^3^: data on significance not available, ICIQ-UI SF at BL: no information, ICIQ-UI SF at 6-month FU: 9 [85]. Significant numbers are in bold. ICIQ-UI SF: International Consultation on Incontinence Questionnaire-Urinary Incontinence Short Form; PFMT: pelvic floor muscle training; RF: radiofrequency; 1h Pad: one-hour pad weight test; sign: significant; BL: baseline; FU: follow-up; SUI: stress urinary incontinence; GSM: genitourinary syndrome of menopause; Er:YAG: Erbium:YAG.

### 4.3. Indication for Laser Therapy

**Intravaginal** Erbium:YAG laser therapy is indicated for women with **mild to moderate SUI** [64,80]. In studies, cure or improvement could be achieved for patients with a one-hour pad weight ≤ 20 g or an ICIQ-UI SF score ≤ 10 at baseline [64,91].

A pilot study showed that **intraurethral** Erbium:YAG laser therapy cured 64% of patients with **severe SUI** [79]. Three additional studies on intraurethral laser therapy were published in 2023. Patients with severe SUI received a combination of intravaginal and intraurethral laser therapy [80]. In a second study, patients with persisting SUI after two courses of unsuccessful intravaginal Erbium:YAG treatments received an intraurethral laser treatment leading to 77% improvement [82]. Elite female athletes after childbirth with unsatisfactory results after three months of pelvic floor muscle training benefited from a combined intravaginal and intraurethral laser therapy [81]. 

The **Erbium:YAG** laser therapy with SMOOTH-mode seemed to be more efficient for **younger early menopausal** women, and the treatment effect lasted longer in this patient group [92]. Other studies confirmed that women with a median age below 50 years [93] or women below 39 years [94] had a significantly higher subjective improvement than older women. Intravaginal Erbium:YAG laser therapy may be an ideal option to treat younger women with SUI after childbirth.

Conversely, **CO_2_ laser** therapy resulted in better outcomes in an **older postmenopausal** patient group with vaginal atrophy [95,96]. Additionally, fractional ablative CO_2_ laser therapy had a higher effect when urinary symptoms started after menopause [97]. Interestingly, the combination of intraurethral and intravaginal Erbium:YAG treatment was found to be more effective in relieving SUI symptoms at postmenopausal state [80].

The difference in the target age groups could be due to the different modes of action of the two lasers. The CO_2_ laser causes tissue damaging with large edema that induces healing processes and the regeneration of the atrophic epithelium, whereas the Erbium:YAG laser targets primarily the contraction of collagen fibers that induces neo-collagenesis and neo-vascularization [98].

Women with a body mass index below 25 kg/m^2^ have a higher chance of improvement after Erbium:YAG laser therapy [91,93]. Erbium:YAG laser therapy can be performed after failed suburethral sling insertion [92].

### 4.4. Safety

Side effects of laser therapy are minor and transient [65]. Studies with the CO_2_ laser reported moderate pain during the procedure, with VAS scores between 3 and 7, occurring mainly at the distal third of the vagina, and 29% of the patients had mild vaginal bleeding after the treatment [87]. No major safety concerns were found [90]. Erbium:YAG laser treatment only very occasionally caused mild pain or a burning sensation during treatment, and in most cases, a topical anesthetic cream was not even necessary [64]. A few patients described increased vaginal discharge after treatment or discomfort in the vaginal and vulvar area. All adverse events were transient and most frequently resolved within 8 days [85]. The insertion of the intraurethral device initially may be painful but can be tolerated with the help of local anesthetics prior to treatment. A survey among practitioners using the Erbium:YAG laser reported only mild to moderate transient adverse events with low frequencies, among them 4.01% vaginal discharge, 3.45% edema, and 1.44% pain during treatment [99].

Application errors cause most of the complications, and therefore education and training are important [66]. Laser settings and the treatment procedure should be practiced before treating patients. Some countries regulate the use of medical lasers and request proof of training. Only doctors with a certificate of competence can bill to insurance for laser treatments. Such qualifications may gain significance in the future [70].

### 4.5. Conclusions—Laser Therapy

Intravaginal laser therapy is effective to treat mild to moderate SUI. For severe SUI, an additional intraurethral treatment may be considered. Subjective and objective cure rates of 30% are described, but the improvement diminished over time, requiring top-up treatments 1 to 2 years after the initial laser therapy. Therapy success was reported for both the non-ablative Erbium:YAG laser with SMOOTH-mode settings and the fractional-ablative CO_2_ laser. An RCT that directly compares the two laser types is not available. Due to different modes of action, the CO_2_ laser treatment seems to be more painful, with a slightly longer recovery time than for the Erbium:YAG laser. Laser treatment can be performed in an ambulatory setting, it is minimally invasive, needs no artificial material, and has only a few temporary side effects. 

Intravaginal laser therapy is an ideal treatment option for young women requiring fast improvement in their symptoms or for postmenopausal women to concurrently treat vulvovaginal atrophy.

## Data Availability

Not applicable.
